# Tunneling of two-dimensional surface polaritons through plasmonic nanoplates on atomically thin crystals

**DOI:** 10.1515/nanoph-2024-0588

**Published:** 2025-01-07

**Authors:** Seojoo Lee, Ji-Hun Kang

**Affiliations:** School of Applied and Engineering Physics, Cornell University, Ithaca, NY 13853, USA; The Institute of Basic Science, Korea University, Seoul 02841, Republic of Korea; Department of Optical Engineering, Kongju National University, Cheonan 31080, Republic of Korea; Department of Future Convergence Engineering, Kongju National University, Cheonan 31080, Republic of Korea; Institute of Application and Fusion for Light, Kongju National University, Cheonan 31080, Republic of Korea

**Keywords:** surface polaritons, two-dimensional materials, graphene plasmons, plasmonic structures, nanogap

## Abstract

We theoretically investigate the tunneling of two-dimensional surface polaritons (2DSPs) through plasmonic nanoplates on atomically thin crystals. By developing an analytic model based on coupled-mode theory, we examine the tunneling efficiency as a function of the plate’s width and height. Our analysis reveals that the tunneling is mediated by evanescent waves induced near the plate edges due to interactions between 2DSPs and the plate, enabling the consistent recoupling of 2DSPs across plates of finite width, regardless of their height. Additionally, we clarify that these evanescent waves not only facilitate tunneling but also induce an anomalous phase shift in the 2DSPs reflected from the plate. This phase shift is shown to play a critical role in the resonant tunneling of 2DSPs through two plates, effectively creating a polariton-assisted resonator in the deep subwavelength regime.

## 1 Introduction

Two-dimensional surface polaritons (2DSPs) are quasiparticles formed by the coupling of photons with material excitations in atomically thin 2D crystals, enabling confinement of electromagnetic waves at deep subwavelength scales [1], [2], [3], [4], [5], [6]. Control of 2DSPs has become pivotal in realizing light manipulation at a reduced low-dimensional space [7], [8], [9], [10], [11], [12]. Similar to the case of light in three-dimensional space, 2DSPs can be controlled by their interaction with a boundary defined by two different media. The edge-reflection of 2DSPs, resulting from the interaction of 2DSPs with a single boundary between the 2D crystal and free-space (i.e., the crystal’s edge) [13], is a prime example of the control of 2DSPs’ propagation by structured 2D crystals [9], [14], [15], [16], [17], [18], and this has been extended later to cases involving abrupt gaps within the crystal itself [19], [20]. Manipulation of 2DSPs using plasmonic nanostructures has also been explored [7]. Controlled radiative coupling of weakly coupled 2DSPs using metal nanostructures on the crystal was investigated [21], [22], and later a detailed study on the reflection of strongly coupled 2DSPs by semi-infinitely wide plasmonic nanoplates was performed [23]. The latter study revealed that a nanoplate can induce strong evanescent waves near its edge through the interaction with incident 2DSPs, resulting in near-total internal reflection of 2DSPs accompanied by an anomalous phase shift in the reflected waves.

In this paper, we theoretically investigate the interaction of 2DSPs with plasmonic nanoplates of finite width and extend the role of the induced evanescent waves to the tunneling of 2DSPs through the plates. By using an analytical model based on coupled-mode theory, we analyze the tunneling efficiency as a function of the plate’s width and height. We also reveal that tunneling is mediated by the evanescent waves induced near the plate edges due to interactions with 2DSPs, enabling consistent recoupling across plates of finite width, irrespective of height. Furthermore, we demonstrate that these evanescent waves not only facilitate tunneling but also induce an anomalous phase shift in the 2DSPs reflected from the plate. This phase shift plays a critical role in the resonant tunneling of 2DSPs through two nanoplates, effectively creating a simple polaritonic resonator in the deep subwavelength regime. These findings provide fundamental insights for the control of 2DSP propagation by using plasmonic meta-structures, offering potential advances in polariton-based applications.

## 2 Theoretical model

**Figure 1:**
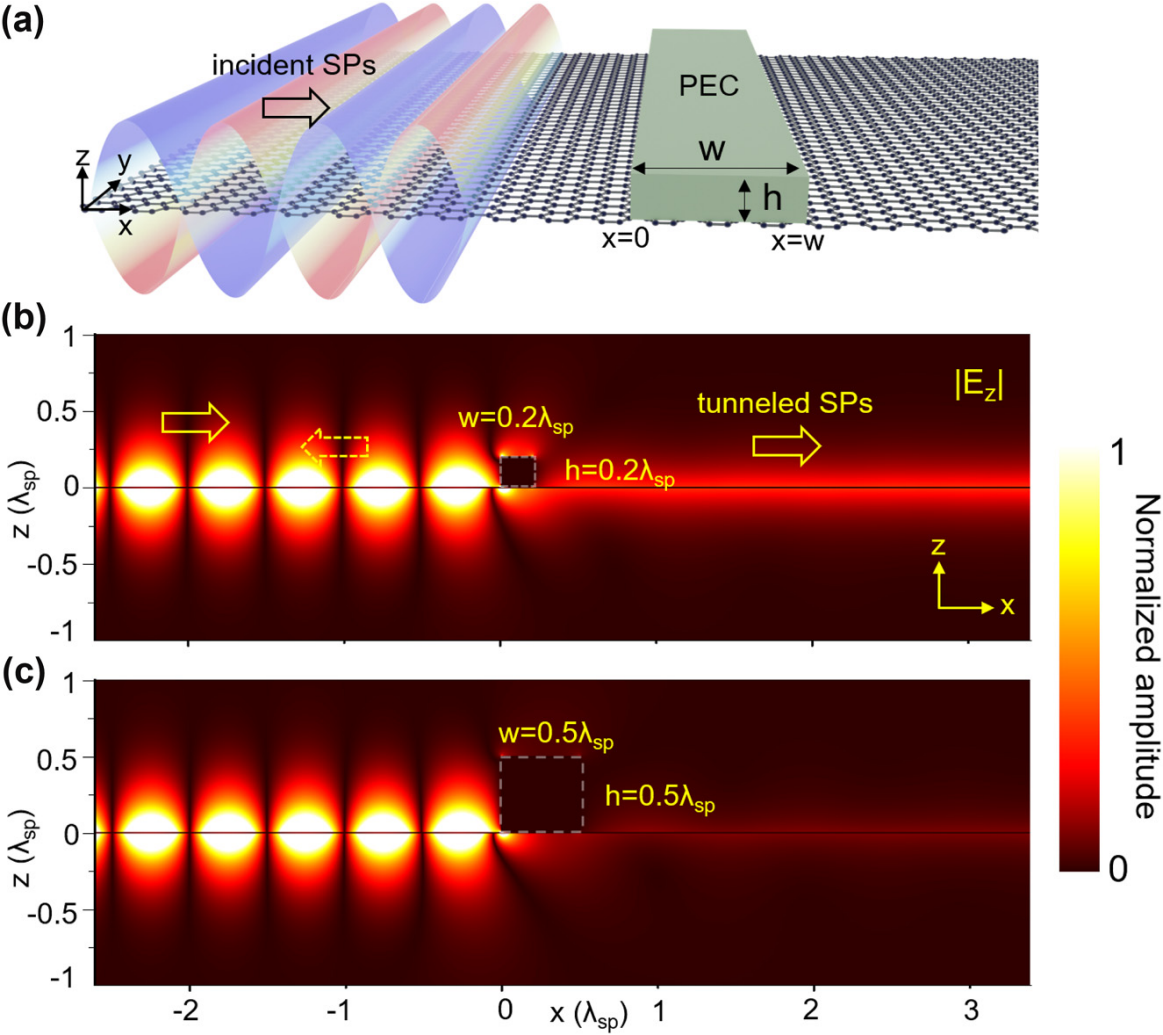
Interaction of 2DSPs with a plasmonic nanoplate. (a) Schematic of the propagation, reflection, and tunneling of 2DSPs through a plasmonic nanoplate. (b, c) Numerically calculated |*E*_*z*_| field maps, demonstrating 2DSP tunneling through plates with (b) *w* = *h* = 0.2*λ*_*sp*_ and (c) *w* = *h* = 0.5*λ*_*sp*_, where *λ*_*sp*_ is the wavelength of 2DSPs. The momentum of 2DSPs is set to *p*_*x*_ = 50*k*_0_. Numerical calculations were performed using the finite-difference time-domain (FDTD) method.

Consider a plasmonic nanoplate of width *w* and height *h*, placed on infinitely wide, infinitesimally thin 2D crystal located at *z* = 0, as shown in Figure 1(a). Strongly surface-bound 2DSPs, excited far to the left of the plate, propagate along the +*x*-direction with momentum *p*_*x*_, which is significantly larger than the photon momentum *k*_0_. For simplicity, we assume that the 2D crystal is lossless and that the plate is perfect electric conductor (PEC), so that 2DSPs do not experience plasmonic damping in the system. The interaction between the incident 2DSPs and the plate involves diffraction of the 2DSPs, resulting in the excitation of all possible orthogonal eigenmodes in the system. The crystal region (*x* ≤ 0 or *x* ≥ *w*) has three eigenmodes: a surface-bound polariton mode $\left|p\right\rangle$ , surface-unbound symmetric, and antisymmetric radiation modes, $\left|s_{k_{z}}\right\rangle$ and $\left|a_{k_{z}}\right\rangle$ , respectively. In the plate region (0 ≤ *x* ≤ *w*), we can separately consider the upper (*z* ≥ *h*) and lower (*z* ≤ 0) regions, with the corresponding eigenmodes $\left|u_{k_{z}}\right\rangle$ and $\left|l_{k_{z}}\right\rangle$ . The real-space representations of the eigenmodes in the crystal region can be expressed as [23]

(1) $$\begin{array}{ll} \left\langle z\left|\right.p\right\rangle & \equiv \frac{z}{\left|z\right|}{\text{e}}^{\text{i}p_{z}|z|},\\ \;\left\langle z\left|\right.\;a_{k_{z}}\right\rangle & \equiv \frac{1}{\sqrt{\pi }}\left(\frac{z}{\left|z\right|}\frac{k_{z}}{p_{z}}\cos\left(k_{z}z\right)+i\sin\left(k_{z}z\right)\right)\text{, }\\ \left\langle z\left|\right.\;s_{k_{z}}\right\rangle & \equiv \frac{1}{\sqrt{\pi }}\cos\left(k_{z}z\right). \end{array}$$

Here, $p_{z}\equiv \sqrt{k_{0}^{2}-p_{x}^{2}}$ , and *k*_*z*_ is the continuous momentum of the radiation modes in the *z*-direction. The eigenmodes in the plate region are plane waves with continuous momentum *k*_*z*_:

(2) $$\begin{array}{ll} \left\langle z\left|\right.\;u_{k_{z}}\right\rangle & \equiv {\text{e}}^{-ik_{z}\left(z-h\right)}+{\text{e}}^{\text{i}k_{z}\left(z-h\right)}\text{,}\\ \left\langle z\left|\right.\;l_{k_{z}}\right\rangle & \equiv {\text{e}}^{-ik_{z}z}+{\text{e}}^{\text{i}k_{z}z}. \end{array}$$

The tunneling of 2DSPs is primarily based on the nonvanishing dependency between eigenmodes from different spaces. For instance, the inner product between $\left|p\right\rangle$ and $\left|u_{k_{z}}\right\rangle$ is nonzero, meaning that $\left|u_{k_{z}}\right\rangle$ can couple with $\left|p\right\rangle$ , and vice versa. As a result, the incident 2DSPs can induce $\left|u_{k_{z}}\right\rangle$ and $\left|l_{k_{z}}\right\rangle$ , which in turn excite 2DSPs at the right crystal region, as demonstrated in Figure 1(b) and (c). Using this macroscopic description of 2DSPs tunneling, we expand the *y*-component of the magnetic field in the crystal region as

(3) $$\begin{array}{ll} \left|H_{y}^{x\leq 0}\left(x\right)\right\rangle & =\left({\text{e}}^{\text{i}p_{x}x}-R{\text{e}}^{-\text{i}p_{x}x}\right)\left|p\right\rangle \\ & \;+\overset{\infty }{\underset{-\infty }{\int }}\text{d}k_{z}\left(\alpha_{k_{z}}\left|s_{k_{z}}\right\rangle +\beta_{k_{z}}\left|a_{k_{z}}\right\rangle \right){\text{e}}^{-\text{i}k_{x}x},\\ \left|H_{y}^{w\leq x}\left(x\right)\right\rangle & =T{\text{e}}^{\text{i}p_{x}\left(x-w\right)}\left|p\right\rangle \\ & \;+\overset{\infty }{\underset{-\infty }{\int }}\text{d}k_{z}\left(\sigma_{k_{z}}\left|s_{k_{z}}\right\rangle +\tau_{k_{z}}\left|a_{k_{z}}\right\rangle \right){\text{e}}^{\text{i}k_{x}\left(x-w\right)}, \end{array}$$

with $k_{x}=\sqrt{k_{0}^{2}-k_{z}^{2}}$ . *R* and *T* represent the reflection and the tunneling coefficients of 2DSPs, respectively, while *α*, *β*, *σ*, and *τ* denote amplitudes for the radiation modes. The reason for referring to *T* as tunneling rather than transmission will be discussed later. In the plate region, the magnetic field can be expressed in terms of continua of $\left|u_{k_{z}}\right\rangle$ and $\left|l_{k_{z}}\right\rangle$ ,

(4) $$\begin{array}{ll} \left|H_{y}^{0\leq x\leq w}\left(x\right)\right\rangle & =\overset{\infty }{\underset{-\infty }{\int }}\text{d}k_{z}\left|u_{k_{z}}\right\rangle \left(A_{k_{z}}{\text{e}}^{\text{i}k_{x}x}+B_{k_{z}}{\text{e}}^{-\text{i}k_{x}x}\right)\\ & \;+\overset{\infty }{\underset{-\infty }{\int }}\text{d}k_{z}\left|l_{k_{z}}\right\rangle \left(C_{k_{z}}{\text{e}}^{\text{i}k_{x}x}+D_{k_{z}}{\text{e}}^{-\text{i}k_{x}x}\right). \end{array}$$

Here, *A*, *B*, *C*, and *D* are amplitudes for the plane wave modes in the plate region. From Eqs. (3) and (4), the corresponding *z*-components of the electric field can be derived directly from Maxwell’s equations. Applying the boundary condition at *x* = 0 yields

(5) $$\begin{array}{ll} & \left(1-R\right)\left|p\right\rangle +\overset{\infty }{\underset{-\infty }{\int }}\text{d}k_{z}\left(\alpha_{k_{z}}\left|s_{k_{z}}\right\rangle +\beta_{k_{z}}\left|a_{k_{z}}\right\rangle \right)\\ & \;=\overset{\infty }{\underset{-\infty }{\int }}\text{d}k_{z}\left[\left(A_{k_{z}}+B_{k_{z}}\right)\left|u_{k_{z}}\right\rangle +\left(C_{k_{z}}+D_{k_{z}}\right)\left|l_{k_{z}}\right\rangle \right],\\ & \left(1+R\right)\left|p\right\rangle -\overset{\infty }{\underset{-\infty }{\int }}\text{d}k_{z}\frac{k_{x}}{p_{x}}\left(\alpha_{k_{z}}\left|s_{k_{z}}\right\rangle +\beta_{k_{z}}\left|a_{k_{z}}\right\rangle \right)\\ & \;=\overset{\infty }{\underset{-\infty }{\int }}\text{d}k_{z}\frac{k_{x}}{p_{x}}\left[\left(A_{k_{z}}-B_{k_{z}}\right)\left|u_{k_{z}}\right\rangle +\left(C_{k_{z}}-D_{k_{z}}\right)\left|l_{k_{z}}\right\rangle \right], \end{array}$$

while the boundary condition at *x* = *w* gives rise to

(6) $$\begin{array}{ll} & T\left|p\right\rangle +\overset{\infty }{\underset{-\infty }{\int }}\text{d}k_{z}\left(\sigma_{k_{z}}\left|s_{k_{z}}\right\rangle +\tau_{k_{z}}\left|a_{k_{z}}\right\rangle \right)\\ & \;=\overset{\infty }{\underset{-\infty }{\int }}\text{d}k_{z}\left[\left(A_{k_{z}}{\text{e}}^{\text{i}k_{x}w}+B_{k_{z}}{\text{e}}^{-\text{i}k_{x}w}\right)\left|u_{k_{z}}\right\rangle \right.\\ & \;\left.+\left(C_{k_{z}}{\text{e}}^{\text{i}k_{x}w}+D_{k_{z}}{\text{e}}^{-\text{i}k_{x}w}\right)\left|l_{k_{z}}\right\rangle \right],\\ & T\left|p\right\rangle +\overset{\infty }{\underset{-\infty }{\int }}\text{d}k_{z}\frac{k_{x}}{p_{x}}\left(\sigma_{k_{z}}\left|s_{k_{z}}\right\rangle +\tau_{k_{z}}\left|a_{k_{z}}\right\rangle \right)\\ & \;=\overset{\infty }{\underset{-\infty }{\int }}\text{d}k_{z}\frac{k_{x}}{p_{x}}\left[\left(A_{k_{z}}{\text{e}}^{\text{i}k_{x}w}-B_{k_{z}}{\text{e}}^{-\text{i}k_{x}w}\right)\left|u_{k_{z}}\right\rangle \right.\\ & \;\left.+\left(C_{k_{z}}{\text{e}}^{\text{i}k_{x}w}-D_{k_{z}}{\text{e}}^{-\text{i}k_{x}w}\right)\left|l_{k_{z}}\right\rangle \right]. \end{array}$$

Since we have 10 undetermined coefficients in our equations, the four equations in Eqs. (5) and (6) may initially appear insufficient. However, it is important to note that these are coupled equations, with the left- and right-hand sides of each equation expressed in different vector spaces with different bases. This means that Eqs. (5) and (6) implicitly contain all 10 required equations for the 10 coefficients, which can be extracted by projecting Eqs. (5) and (6) onto the eigenmodes of the system. Specifically, we can project the two equations in Eq. (5) onto $\left|u_{k_{z}}\right\rangle$ and $\left|l_{k_{z}}\right\rangle$ to obtain four coupled equations and project the remaining equations in Eq. (6) onto $\left|p\right\rangle$ , $\left|s_{k_{z}}\right\rangle$ , and $\left|a_{k_{z}}\right\rangle$ to obtain additional six coupled equations [24]. The complexity of the coupled integral equations arises from the nonzero dependencies between the four eigenmodes with continuous momenta. Here, we apply the first Born approximation (BA), which, in our case, corresponds to neglecting dependency between eigenmodes with different continuous momenta. Under the BA, all dependencies, determining coupling between the radiation modes in the crystal region and the plane wave modes in the plate region, can be represented in terms of the Dirac’s delta function. This significantly reduces the complexity of the coupled integral equations by eliminating integral terms in eight of the equations. After some manipulations, one can show that the coupled equations reduce to two equations [24],

(7) $$\begin{array}{ll} \left(1+R\right)\left\langle p\left|\right.p\right\rangle & =\overset{\infty }{\underset{-\infty }{\int }}\text{d}k_{z}\frac{k_{x}}{p_{x}}\left[\left(A_{k_{z}}-B_{k_{z}}\right)\left\langle p\left|\right.\;u_{k_{z}}\right\rangle \right.\\ & \;\left.+\left(C_{k_{z}}-D_{k_{z}}\right)\left\langle p\left|\right.\;l_{k_{z}}\right\rangle \right],\\ T\left\langle p\left|\right.p\right\rangle & =\overset{\infty }{\underset{-\infty }{\int }}\text{d}k_{z}\left[\frac{k_{x}}{p_{x}}\left(A_{k_{z}}{\text{e}}^{\text{i}k_{x}w}-B_{k_{z}}{\text{e}}^{-\text{i}k_{x}w}\right)\right.\\ & \;\times \left\langle p\left|\right.\;u_{k_{z}}\right\rangle +\left(C_{k_{z}}{\text{e}}^{\text{i}k_{x}w}-D_{k_{z}}{\text{e}}^{-\text{i}k_{x}w}\right)\\ & \left.\;\times \left\langle p\left|\right.\;l_{k_{z}}\right\rangle \right]. \end{array}$$

The four coefficients *A*, *B*, *C*, and *D* can be determined by the following matrix equation,

(8) $$MV=\left[\begin{array}{cc} M_{11} & M_{12}\\ M_{21} & M_{22} \end{array}\right]V=F,$$

where the 2 × 2 submatrix *M*_*mn*_ and 4 × 1 column vectors *V* and *F* are defined as

(9) $$\begin{array}{cc} M_{ij} & \equiv \left[\begin{array}{cc} m_{i,j}+2\delta_{ij} & -m_{i,j}+2\delta_{ij}\\ \left(-m_{i,j}+2\delta_{ij}\right){\text{e}}^{\text{i}k_{x}w} & \left(m_{i,j}+2\delta_{ij}\right){\text{e}}^{-\text{i}k_{x}w} \end{array}\right],\\ \;V^{T} & \equiv \left[\begin{array}{cccc} A_{k_{z}} & B_{k_{z}} & C_{k_{z}} & D_{k_{z}} \end{array}\right],\\ F^{T} & \equiv \frac{1}{2\pi }\left[\begin{array}{cccc} \left(1-R\right)\left\langle u_{k_{z}}\left|\right.p\right\rangle & T\left\langle u_{k_{z}}\left|\right.p\right\rangle & \left(1-R\right)\left\langle l_{k_{z}}\left|\right.p\right\rangle & T\left\langle l_{k_{z}}\left|\right.p\right\rangle \end{array}\right], \end{array}$$

with *δ*_*ij*_ the Kronecker delta. The matrix element *m*_*i,j*_ is given by

(10) $$\begin{array}{ll} m_{i,j} & \equiv \frac{1}{4\pi }\left[W_{v_{i}\leftarrow s}W_{s\leftarrow v_{j}}+{\left(1-\frac{k_{z}^{2}}{p_{z}^{2}}\right)}^{-1}W_{v_{i}\leftarrow a}W_{a\leftarrow v_{j}}\right]\;,\\ & v_{1}\equiv u,\;\;v_{2}\equiv l. \end{array}$$

The dimensionless factor *W*_*b←a*_ represents the coupling between two eigenmodes $\left|a\right\rangle$ and $\left|b\right\rangle$ under the BA. For instance, $W_{s\leftarrow u}\equiv \int_{-\infty }^{\infty }\text{d}k_{\zeta }\left\langle s_{k_{z}}\left|\right.\;u_{k_{\zeta }}\right\rangle =\int_{-\infty }^{\infty }\text{d}k_{\zeta }\int_{-\infty }^{\infty }dz\left\langle s_{k_{z}}\left|\right.\;z\right\rangle \left\langle z\left|\right.\;u_{k_{\zeta }}\right\rangle \approx \sqrt{\pi }\left({\text{e}}^{\text{i}k_{z}h}+{\text{e}}^{-\text{i}k_{z}h}\right)$ . It is now straightforward to determine the four coefficients from Eq. (8), and substituting them into Eq. (7) yields the reflection and tunneling coefficients as

(11) $$R=\frac{I_{1}^{2}-1-I_{2}^{2}}{{\left(I_{1}+1\right)}^{2}-I_{2}^{2}},\;\;T=\frac{-2I_{2}}{{\left(I_{1}+1\right)}^{2}-I_{2}^{2}}.$$

The two coupling strengths, *I*_1_ and *I*_2_, are given by

(12) $$\begin{array}{ll} I_{j} & \equiv \frac{1}{2\pi }\frac{1}{\left\langle p\left|\right.p\right\rangle }\overset{\infty }{\underset{-\infty }{\int }}\text{d}k_{z}\left[\left\langle p\left|\right.\;u_{k_{z}}\right\rangle P_{1j}+\left\langle p\left|\right.\;l_{k_{z}}\right\rangle P_{3j}\right],\\ P_{ij} & \equiv \frac{k_{x}}{p_{x}}\left[\left\langle u_{k_{z}}\left|\right.\;p\right\rangle \left(n_{i,j}-n_{i+1,j}\right)\right.\\ & \;+\left.\left\langle l_{k_{z}}\left|\right.\;p\right\rangle \left(n_{i,j+2}-n_{i+1,j+2}\right)\right], \end{array}$$

where *n*_*i,j*_ are the elements of matrix *N* ≡ *M* ^−1^.

## 3 Results and discussion

Before proceeding with further discussions, it should be noted that we have defined *T* as the tunneling coefficient rather than as a transmission coefficient. This distinction is motivated by the strong momentum mismatch between 2DSPs and the eigenmodes in the nanoplate region, which suppresses the radiative coupling of $\left|p\right\rangle$ to $\left|u_{k_{z}}\right\rangle$ and $\left|l_{k_{z}}\right\rangle$ . A quantitative account of the coupling between these two modes involves their inner product. Specifically, the coupling between $\left|p\right\rangle$ and $\left|l_{k_{z}}\right\rangle$ , for instance, is governed by the factor ${\left|\left\langle p\left|\right.l_{k_{z}}\right\rangle \right|}^{2}={\left(2ip_{z}/\left(k_{z}^{2}-p_{z}^{2}\right)\right)}^{2}$ . This factor possesses very small portion of radiative momentum range (0 ≤ *k*_*z*_ ≤ *k*_0_) for strongly confined 2DSPs (*p*_*x*_ >> *k*_0_), making the radiative coupling of 2DSPs negligible. A significance of this is that the induced electric fields near the left edge of the plate, as shown in Figure 1(b) and (c) near *x* = 0, are mostly evanescent waves.

**Figure 2:**
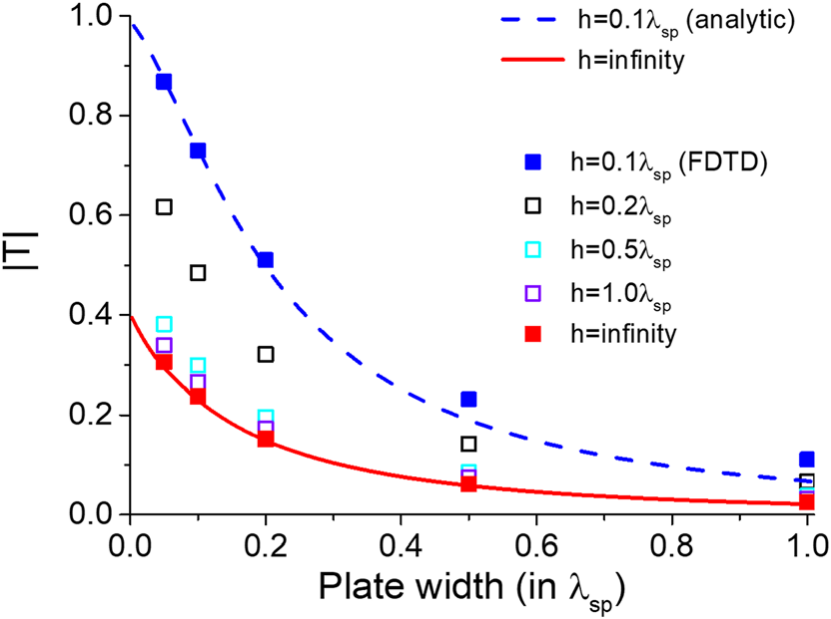
Analytically (curves) and numerically (squares) calculated tunneling amplitudes for 2DSPs as a function of nanoplate width and height. Varying width and height of the plate. We set *p*_*x*_ = 50*k*_0_.

Figure 2 shows the tunneling behavior of 2DSPs through a nanoplate, as influenced by the plate’s width and height. The results from our analytic theory show good agreement with the FDTD calculations, with minor discrepancies observed for wider plates, which we attribute to the limitation of the BA. The tunneling efficiency, defined by |*T*|, decays monotonically as width and height of the plate increase. However, while the tunneling efficiency approaches zero as the width tends to infinity, 2DSPs can still tunnel through a nanoplate of infinite height, provided the width remains finite. This behavior occurs because the evanescent waves can always be induced near the bottom corners (*x* = 0 and *w* with *z* = 0) of a finite-width plate and subsequently recouple to 2DSPs in the right crystal region. The “guaranteed” tunneling efficiency for a plate with finite width can be obtained by excluding the contribution from $\left|u_{k_{z}}\right\rangle$ , which simplifies the coupling strengths in Eq. (12) as

(13) $$\begin{array}{ll} I_{1}^{h\to \infty } & =\frac{1}{2\pi }\frac{1}{\left\langle p\left|\right.p\right\rangle }\overset{\infty }{\underset{-\infty }{\int }}\text{d}k_{z}\frac{k_{x}}{p_{x}}{\left|\left\langle l_{k_{z}}\left|\right.p\right\rangle \right|}^{2}\\ & \;\times \frac{\left(m_{2,2}+2\right){\text{e}}^{-\text{i}k_{x}w}-\left(m_{2,2}-2\right){\text{e}}^{\text{i}k_{x}w}}{{\left(m_{2,2}+2\right)}^{2}{\text{e}}^{-\text{i}k_{x}w}-{\left(m_{2,2}-2\right)}^{2}{\text{e}}^{\text{i}k_{x}w}},\\ I_{2}^{h\to \infty } & =-\frac{1}{2\pi }\frac{1}{\left\langle p\left|\right.p\right\rangle }\overset{\infty }{\underset{-\infty }{\int }}\text{d}k_{z}\frac{k_{x}}{p_{x}}{\left|\left\langle l_{k_{z}}\left|\right.p\right\rangle \right|}^{2}\\ & \;\times \frac{4}{{\left(m_{2,2}+2\right)}^{2}{\text{e}}^{-\text{i}k_{x}w}-{\left(m_{2,2}-2\right)}^{2}{\text{e}}^{\text{i}k_{x}w}}, \end{array}$$

where $m_{2,2}={\left(4\pi \right)}^{-1}\left[W_{l\leftarrow s}W_{s\leftarrow l}+{\left(1-k_{z}^{2}/p_{z}^{2}\right)}^{-1}W_{l\leftarrow a}W_{a\leftarrow l}\right]\approx \left(p_{z}^{2}-2k_{z}^{2}\right){\left(p_{z}^{2}-k_{z}^{2}\right)}^{-1}$ . The tunneling efficiency obtained from Eq. (13) is represented by the red curve in Figure 2, demonstrating good agreement with numerical FDTD results. Notably, parts of the integrands in Eq. (13) resemble the transmission and reflection coefficients for a plane wave interacting with a slab of finite thickness [25], highlighting the intuitive notion that the integrands implicate multiple reflections of $\left|l_{k_{z}}\right\rangle$ by two boundaries at *x* = 0 and *x* = *w*. Furthermore, it is worth noting that the tunneling efficiency for *h* = 0.1*λ*_*sp*_ is more than twice as high as that for an infinite height, implying that the interaction between $\left|u_{k_{z}}\right\rangle$ and $\left|l_{k_{z}}\right\rangle$ , represented by the off-diagonal submatrices *M*_12_ and *M*_21_ in Eq. (8), is non-negligible and plays a notable role in the tunneling process. A quantitative account for this interaction, however, will be deferred to future work.

**Figure 3:**
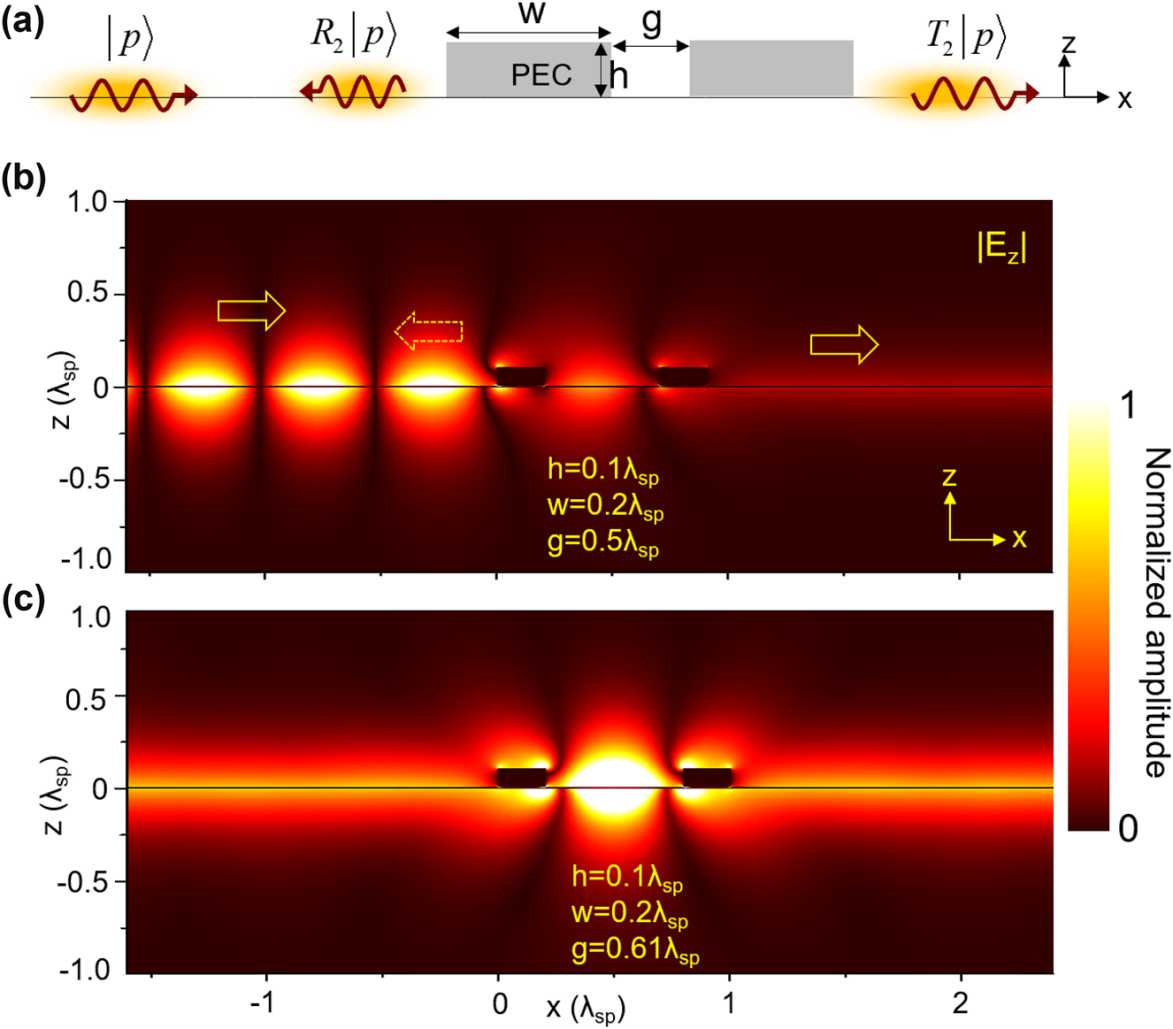
Resonant tunneling of 2DSPs through a two identical nanoplates. (a) Schematic of the two nanoplate system with gap size *g* between the plates. (b, c) FDTD-calculated |*E*_*z*_| field maps for (b) *g* = 0.5*λ*_*sp*_ and (c) *g* = 0.61*λ*_*sp*_, demonstrating the effect of gap size on resonant tunneling. In both cases, the nanoplate height is set to *h* = 0.1*λ*_*sp*_, width to *w* = 0.2*λ*_*sp*_, and momentum to *p*_*x*_ = 50*k*_0_.

So far, we have discussed the tunneling properties of 2DSPs through a plasmonic nanoplate, in terms of tunneling efficiency as functions of the plate’s height and width. Considering that a nanoplate acts as a barrier to 2DSPs, it is possible to expect resonant tunneling of 2DSPs when additional plates are introduced, similar to resonant quantum tunneling through multiple barriers. The simplest configuration for this consists of two identical plates on the 2D crystal, separated by a gap size *g*, as illustrated in Figure 3(a). Shown in Figure 3(b) and (c) are FDTD-calculated maps of |*E*_*z*_| for the two-plate configuration, with each plate having a fixed height of 0.1*λ*_*sp*_ and width of 0.2*λ*_*sp*_. Figure 3(b) shows the field distribution when the gap size is *g* = 0.5*λ*_*sp*_. Here, clear interference fringes of 2DSPs are visible in the left crystal region, indicating that most of the incident 2DSPs are reflected by the plates. In contrast, Figure 3(c) illustrates the field distribution when the gap size is increased to 0.61*λ*_*sp*_. In this configuration, the fringes disappear, and distinctive 2DSPs are observed in the right crystal region, suggesting that the reflection is suppressed and most of the incident 2DSPs successfully tunnel through the plates. This demonstrates that 2DSPs can resonantly tunnel through two nanoplates when specific geometric conditions are met.

To achieve a thorough analytic account of the resonant tunneling of 2DSPs, the same eigenmode expansions used in Eq. (1) can be applied to the five distinct regions formed by the two nanoplates. However, as we have previously discussed, the radiation modes are mostly evanescent waves that decay exponentially as the distance from the plate increases. This suggests that the interaction between the two plates is primarily mediated by 2DSPs, especially when the plates are not very close to each other. In this sense, we apply a systematic approximation that excludes the contribution of radiation modes to the interaction. This approach simplifies the system to a simple cavity where only multiple reflections of 2DSPs between the plates are considered. With this cavity model, the tunneling and reflection coefficients, *T*_2_ and *R*_2_, can be derived as

(14) $$T_{2}=\frac{T^{2}{\text{e}}^{\text{i}p_{x}g}}{1-R^{2}{\text{e}}^{2\text{i}p_{x}g}},\;\;R_{2}=R+\frac{T^{2}{\text{e}}^{2\text{i}p_{x}g}}{1-R^{2}{\text{e}}^{2\text{i}p_{x}g}}.$$

**Figure 4:**
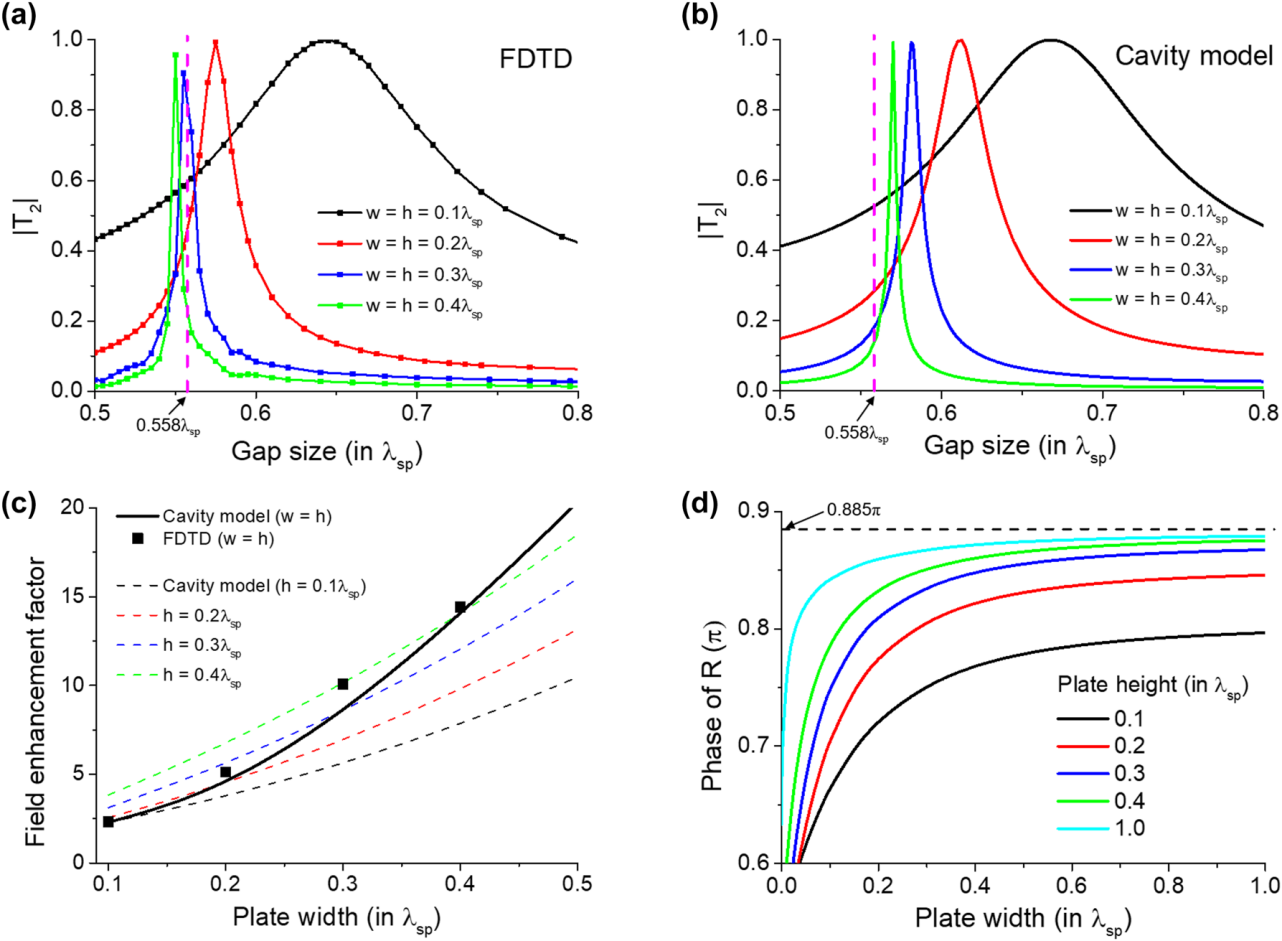
Resonant tunneling of 2DSPs as a function of gap size *g*. (a) and (b) The tunneling amplitude |*T*_2_| calculated using (a) FDTD simulations and (b) the cavity model from Eq. (14). In (a) and (b), the vertical magenta dashed lines indicate the saturating gap size *g* = 0.558*λ*_*sp*_, predicted by the cavity model with arg(*R*) = 0.885*π* as denoted in (d). (c) Enhancement factor of the electric field inside the cavity at the resonance. The enhancement factor is defined by normalizing |*E*_*z*_(*z* = 0^+^)| at the center of the gap by the field of the incident 2DSPs at *z* = 0^+^. (d) Analytically calculated phase of the reflection coefficient *R* in Eq. (11) as a function of nanoplate width *w*. The momentum of 2DSPs is set to *p*_*x*_ = 50*k*_0_ for all calculations.

Figure 4(a) and (b) show the results of 2DSP tunneling through two nanoplates, calculated using the cavity model and FDTD simulations, respectively, as a function of the gap size for exemplary plate widths and heights. Both results demonstrate that the resonant tunneling occurs at specific gap sizes, with the resonance gap size decreasing as the width and height of the plates increase. Notably, this resonance behavior tends to approach a saturation gap size as the plate dimensions increase. Here, we emphasize two key points. First, the result highlights the ability to tune the resonance gap size by adjusting the plate dimensions. This tunability, coupled with the strong confinement of the polariton, presents opportunities for applications requiring ultra-small resonators and resonant light concentrations in deep subwavelength regimes. An immediate relevant physical quantity observable in our structure is the resonant enhancement of electric field. Figure 4(c) presents the enhancement factor of the electric field |*E*_*z*_| at the center of the cavity (gap) at resonance. The enhancement factor is calculated by normalizing |*E*_*z*_(*z* = 0^+^)| at the center of the gap by the electric field of the incident 2DSPs. The results indicate that our structure enables strong field enhancement within the cavity, with the electric field increasing as the nanoplate dimensions grow. This demonstrates the capability of the nanoplate configuration to act as a polariton-assisted ultra-small plasmonic resonator, achieving strong electric field confinement in the deep subwavelength regime. In our theoretical demonstration, we set *p*_*x*_ = 50*k*_0_, which corresponds to a gap size of 0.6*λ*_*sp*_ being roughly on the order of *λ*_0_/80. For instance, in the case of an excited graphene plasmon with a free-space wavelength *λ*_0_ = 6 μm and a plasmon wavelength of 120 nm, the gap size would be just about 70 nm, demonstrating the emergence of resonance at a deep subwavelength scale. Second, both the FDTD and cavity model results indicate that the tunneling efficiency can reach nearly 100 %, supporting our claim again that the radiative losses are negligible and that the tunneling is mediated by evanescent waves. This near-complete transmission suggests that the system operates effectively as an effective polaritonic coupler, where the strongly confined nature of the polariton field allows energy transfer across the nanoplates with minimal radiative loss. This unique characteristic may be harnessed in high-efficiency, low-loss devices for applications in nanophotonics and plasmonics. However, it should be noted that the system’s losses can have a noticeable impact on the amplitude of resonant tunneling [24].

In terms of the resonance gap size behavior, we note that the cavity model provides a qualitative prediction of this behavior, by indicating that the resonance can appear when the gap size satisfies the condition *g* = (*mπ* − *ϕ*)/*p*_*x*_ with non-negative integer *m* and *p*_*x*_ ≡ 2*π*/*λ*_*sp*_. Here, *ϕ* ≡ arg(*R*) is the phase of the reflection coefficient, as shown in Figure 4(d) for various values of *h*. The phase shift is shown to be positive, so the zeroth resonance (*m* = 0) is not allowed. On the other hand, in principle, the first resonance (*m* = 1) should be allowed. However, we found numerically that the first resonance is not always guaranteed, presumably due to strong interaction between the radiation modes, as *m* = 1 requires two nanoplates to be very close, with *g* ∼ 0.1*λ*_*sp*_. A deeper physical understanding is required to examine this behavior, but we believe that this should be addressed in a future work. Therefore, here we focus on the second resonance (*m* = 2). Figure 4(d) shows that the phase increases with the width and height of the plate. Also, the phase gradually approaches 0.885*π*, which is a value predicted by the previous study [23]. This trend in the phase explains both the reduction in the resonance gap size observed in Figure 4(a) and (b) with increasing plate’s dimensions, and the saturating behavior. With *ϕ* = 0.885*π*, the saturated gap size is predicted to be approximately *g* ≈ 0.558*λ*_*sp*_, as indicated by the vertical dashed lines in Figure 4(a) and (b). Overall, the results from the cavity model and FDTD calculations show reasonable agreement in both the resonance gap size and the enhancement factor, with about 5 ∼ 10 % discrepancies. The discrepancies are attributed to the ignored contribution of the radiation modes in the simple cavity model and the limitations of the first BA. More specifically, the limitation of the first BA mainly comes from the reduced contribution from the radiation modes. Specifically, the first BA neglects the coupling between the radiation modes in the crystal region and the plane wave in the nanoplate region when the momenta of the two modes differ. This effectively corresponds to a change in the excitation of the evanescent waves near the plate, leading to changes in the reflection phase shift [23] and the resonance gap size. However, we stress that the qualitative agreement between the two methods validates the cavity model’s effectiveness in capturing the essential physics of 2DSP resonant tunneling through multiple nanoplates.

## 4 Conclusions

In this paper, we theoretically investigated the tunneling of two-dimensional surface polaritons (2DSPs) through plasmonic nanoplates on atomically thin crystals. Using an analytical model and FDTD numerical simulations, we demonstrated that evanescent waves near the plate edges mediate the tunneling process, enabling consistent 2DSP recoupling across plates of finite width, irrespective of height. We also revealed that these evanescent waves induce an anomalous phase shift, which is dependent on the plate’s height and width. The phase shift is shown to facilitate resonant tunneling in configurations with two plates, effectively forming a subwavelength polaritonic resonator. These insights offer a pathway not only for precise control of 2DSP propagation in low-dimensional systems by using plasmonic meta-structures but also for polariton-assisted resonant light concentration in a deep subwavelength regime, providing potential applications in ultra-small nanostructure-integrated devices. Moreover, the gate-tunability of 2D crystals can be leveraged to design ultra-compact, gate-tunable polaritonic modulators by tuning the resonant tunneling conditions. This concept can be readily explored using existing nanofabrication techniques and scanning near-field optical microscopy (SNOM) setups by measuring the gate-dependent reflection of 2DSPs.

## Supplementary Material

Supplementary Material Details
