# Supplementary material for: Tunneling of two-dimensional surface polaritons through plasmonic nanoplates on atomically thin crystals
Source: Nanophotonics. 2025 Jan 7;14(8):1185–92. doi: 10.1515/nanoph-2024-0588 (PMC12019953; doi:10.1515/nanoph-2024-0588)
Supplement: Supplementary file 1 — Supplementary Material Details [file j_nanoph-2024-0588_suppl_001.pdf]

Supplementary Material for

"Tunneling of two-dimensional surface polaritons through plasmonic nano-plates on atomically thin crystals"

Seojoo Lee[1,2] and Ji-Hun Kang[3,4,5*]

[1] School of Applied and Engineering Physics, Cornell University, Ithaca, New York 13853, USA

[2] The Institute of Basic Science, Korea University, Seoul 02841, Republic of Korea

[3] Department of Optical Engineering, Kongju National University, Cheonan 31080, Republic of Korea

[4] Department of Future Convergence Engineering, Kongju National University, Cheonan 31080, Republic of Korea

[5] Institute of Application and Fusion for Light, Kongju National University, Cheonan 31080, Republic of Korea

Corresponding author: *jihunkang@kongju.ac.kr

1. Dependencies between the eigenmodes

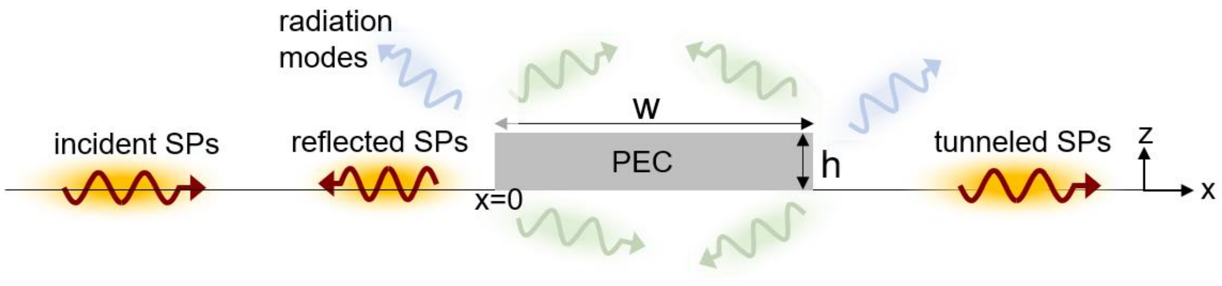

Fig. S1. Schematic of the propagation, reflection and tunneling of 2DSPs through a plasmonic nano-plate, highlighting the excitation of the radiation modes.

In the main text, we used five eigenmodes to describe the interaction between two-dimensional surface polaritons (2DSPs) [1-4] and radiation modes [5] in the 2D crystal region, and the plane wave modes in the nano-plate region, as shown in Fig. S1. The real-space representations of the eigenmodes are as follows [3, 4]:

$$\begin{aligned}&\langle z|p\rangle=\frac{z}{|z|}e^{ip_z|z|},\ \langle z|a_{k_z}\rangle=\frac{1}{\sqrt{\pi}}\left(\frac{z}{|z|}\frac{k_z}{p_z}\cos(k_z z)+i\sin(k_z z)\right),\ \langle z|s_{k_z}\rangle=\frac{1}{\sqrt{\pi}}\cos(k_z z),\\&\langle z|u_{k_z}\rangle=e^{-ik_z(z-h)}+e^{ik_z(z-h)}\left(\text{for } z\ge h\right),\ \langle z|l_{k_z}\rangle=e^{-ik_z z}+e^{ik_z z}\left(\text{for } z\le 0\right).\end{aligned}\tag{S1}$$

Detailed dependencies between the eigenmodes can be directly calculated by projecting one eigenmode to another. Specifically, orthogonality of the radiation modes and the planewave modes can be written as

$$\begin{aligned}&\langle u_{k_\zeta}|u_{k_z}\rangle=2\pi\left[\delta(k_z-k_\zeta)+\delta(k_z+k_\zeta)\right],\ \langle l_{k_\zeta}|l_{k_z}\rangle=2\pi\left[\delta(k_z-k_\zeta)+\delta(k_z+k_\zeta)\right]\\&\langle a_{k_\zeta}|a_{k_z}\rangle=\left(1-\frac{k_z^2}{p_z^2}\right)\left[\delta(k_z-k_\zeta)-\delta(k_z+k_\zeta)\right],\ \langle s_{k_\zeta}|s_{k_z}\rangle=\delta(k_z+k_\zeta)+\delta(k_z-k_\zeta),\end{aligned}\tag{S2}$$

while non-orthogonal dependencies can be obtained as

$$\begin{aligned}
&\left\langle u_{k_\zeta} \middle| p \right\rangle = -\frac{2ip_z}{k_\zeta^2 - p_z^2} e^{ip_z h}, \quad \left\langle l_{k_z} \middle| p \right\rangle = \frac{2ip_z}{k_z^2 - p_z^2}, \\
&\left\langle u_{k_\zeta} \middle| a_{k_z} \right\rangle = \sqrt{\pi}\left(\frac{k_z}{p_z}\cos\left(k_z h\right) + i\sin\left(k_z h\right) \right)\left[\delta\left(k_z - k_\zeta\right) + \delta\left(k_z + k_\zeta\right) \right] \\
&+ \frac{2i}{\sqrt{\pi}} \frac{k_z}{k_z^2 - k_\zeta^2}\left[\cos\left(k_z h\right) + \frac{k_z}{p_z} i\sin\left(k_z h\right) \right], \\
&\left\langle l_{k_\zeta} \middle| a_{k_z} \right\rangle = -\frac{1}{\sqrt{\pi}} \frac{2ik_z}{k_z^2 - k_\zeta^2} - \sqrt{\pi}\frac{k_z}{p_z}\left[\delta\left(k_z - k_\zeta\right) + \delta\left(k_z + k_\zeta\right) \right], \\
&\left\langle u_{k_\zeta} \middle| s_{k_z} \right\rangle = \sqrt{\pi}\cos\left(k_z h\right)\left[\delta\left(k_z - k_\zeta\right) + \delta\left(k_z + k_\zeta\right) \right] - \frac{2}{\sqrt{\pi}}\sin\left(k_z h\right)\frac{k_z}{k_z^2 - k_\zeta^2}, \\
&\left\langle l_{k_\zeta} \middle| s_{k_z} \right\rangle = \sqrt{\pi}\left[\delta\left(k_z - k_\zeta\right) + \delta\left(k_z + k_\zeta\right) \right].
\end{aligned} \tag{S3}$$

2. Full descriptions of 10 coupled integral equations

In Eq. (4) and (5) in the main text, we obtained 4 coupled integral equations written in different vector spaces with different bases. However, Eq. (4) and (5) possess all required 10 equations that can be extracted by applying the projection of Eq. (4) and (5) onto the eigenmodes of the system. Specifically, we can project the two equations in Eq. (4) onto $\left| u_{k_z} \right\rangle$ and $\left| l_{k_z} \right\rangle$ to obtain four coupled equations as

$$\begin{aligned}
&(1-R)\left\langle u_{k_\zeta} \middle| p \right\rangle + \int_{-\infty}^{\infty} dk_z \left(\alpha_{k_z}\left\langle u_{k_\zeta} \middle| s_{k_z} \right\rangle + \beta_{k_z}\left\langle u_{k_\zeta} \middle| a_{k_z} \right\rangle \right) = \int_{-\infty}^{\infty} dk_z \left(A_{k_z} + B_{k_z} \right)\left\langle u_{k_\zeta} \middle| u_{k_z} \right\rangle, \\
&(1-R)\left\langle l_{k_\zeta} \middle| p \right\rangle + \int_{-\infty}^{\infty} dk_z \left(\alpha_{k_z}\left\langle l_{k_\zeta} \middle| s_{k_z} \right\rangle + \beta_{k_z}\left\langle l_{k_\zeta} \middle| a_{k_z} \right\rangle \right) = \int_{-\infty}^{\infty} dk_z \left(C_{k_z} + D_{k_z} \right)\left\langle l_{k_\zeta} \middle| l_{k_z} \right\rangle, \\
&T\left\langle u_{k_\zeta} \middle| p \right\rangle + \int_{-\infty}^{\infty} dk_z \left(\sigma_{k_z}\left\langle u_{k_\zeta} \middle| s_{k_z} \right\rangle + \tau_{k_z}\left\langle u_{k_\zeta} \middle| a_{k_z} \right\rangle \right) = \int_{-\infty}^{\infty} dk_z \left\langle u_{k_\zeta} \middle| u_{k_z} \right\rangle\left(A_{k_z} e^{ik_x w} + B_{k_z} e^{-ik_x w} \right), \\
&T\left\langle l_{k_\zeta} \middle| p \right\rangle + \int_{-\infty}^{\infty} dk_z \left(\sigma_{k_z}\left\langle l_{k_\zeta} \middle| s_{k_z} \right\rangle + \tau_{k_z}\left\langle l_{k_\zeta} \middle| a_{k_z} \right\rangle \right) = \int_{-\infty}^{\infty} dk_z \left\langle l_{k_\zeta} \middle| l_{k_z} \right\rangle\left(C_{k_z} e^{ik_x w} + D_{k_z} e^{-ik_x w} \right),
\end{aligned} \tag{S4}$$

and project the remaining equations in Eq. (5) onto $\left| p \right\rangle$, $\left| s_{k_z} \right\rangle$, and $\left| a_{k_z} \right\rangle$ to have additional six coupled equations such that

$$
\begin{aligned}
&p_x\left(1+R\right)\langle p|p\rangle=\int_{-\infty}^{\infty}dk_zk_x\left[\left(A_{k_z}-B_{k_z}\right)\langle p|u_{k_z}\rangle+\left(C_{k_z}-D_{k_z}\right)\langle p|l_{k_z}\rangle\right],\\
&p_xT\langle p|p\rangle=\int_{-\infty}^{\infty}dk_zk_x\left[\left(A_{k_z}e^{ik_xw}-B_{k_z}e^{-ik_xw}\right)\langle p|u_{k_z}\rangle+\left(C_{k_z}e^{ik_xw}-D_{k_z}e^{-ik_xw}\right)\langle p|l_{k_z}\rangle\right],\\
&-\int_{-\infty}^{\infty}dk_zk_x\alpha_{k_z}\langle s_{k_\zeta}|s_{k_z}\rangle=\int_{-\infty}^{\infty}dk_zk_x\left[\left(A_{k_z}-B_{k_z}\right)\langle s_{k_\zeta}|u_{k_z}\rangle+\left(C_{k_z}-D_{k_z}\right)\langle s_{k_\zeta}|l_{k_z}\rangle\right],\\
&-\int_{-\infty}^{\infty}dk_zk_x\beta_{k_z}\langle a_{k_\zeta}|a_{k_z}\rangle=\int_{-\infty}^{\infty}dk_zk_x\left[\left(A_{k_z}-B_{k_z}\right)\langle a_{k_\zeta}|u_{k_z}\rangle+\left(C_{k_z}-D_{k_z}\right)\langle a_{k_\zeta}|l_{k_z}\rangle\right],\\
&\int_{-\infty}^{\infty}dk_zk_x\sigma_{k_z}\langle s_{k_\zeta}|s_{k_z}\rangle\\
&=\int_{-\infty}^{\infty}dk_zk_x\left[\left(A_{k_z}e^{ik_xw}-B_{k_z}e^{-ik_xw}\right)\langle s_{k_\zeta}|u_{k_z}\rangle+\left(C_{k_z}e^{ik_xw}-D_{k_z}e^{-ik_xw}\right)\langle s_{k_\zeta}|l_{k_z}\rangle\right],\\
&\int_{-\infty}^{\infty}dk_zk_x\tau_{k_z}\langle a_{k_\zeta}|a_{k_z}\rangle\\
&=\int_{-\infty}^{\infty}dk_zk_x\left[\left(A_{k_z}e^{ik_xw}-B_{k_z}e^{-ik_xw}\right)\langle a_{k_\zeta}|u_{k_z}\rangle+\left(C_{k_z}e^{ik_xw}-D_{k_z}e^{-ik_xw}\right)\langle a_{k_\zeta}|l_{k_z}\rangle\right].
\end{aligned}
\tag{S5}
$$

3. First Born approximation

Under the first Born approximation (BA), all dependency between the eigenmodes are suppressed when two momenta of the eigenmodes differ. Then, $\langle u_{k_\zeta}|a_{k_z}\rangle,\langle l_{k_\zeta}|a_{k_z}\rangle$, and $\langle u_{k_\zeta}|s_{k_z}\rangle$ in Eq. (S3) become

$$
\begin{aligned}
&\langle u_{k_\zeta}|a_{k_z}\rangle\approx\sqrt{\pi}\left(\frac{k_z}{p_z}\cos\left(k_zh\right)+i\sin\left(k_zh\right)\right)\left[\delta\left(k_z-k_\zeta\right)+\delta\left(k_z+k_\zeta\right)\right],\\
&\langle l_{k_\zeta}|a_{k_z}\rangle\approx-\sqrt{\pi}\,\frac{k_z}{p_z}\left[\delta\left(k_z-k_\zeta\right)+\delta\left(k_z+k_\zeta\right)\right],\\
&\langle u_{k_\zeta}|s_{k_z}\rangle\approx\sqrt{\pi}\cos\left(k_zh\right)\left[\delta\left(k_z-k_\zeta\right)+\delta\left(k_z+k_\zeta\right)\right].
\end{aligned}
\tag{S6}
$$

One can readily find that the coupled integral equations in Eq. (S4) reduce to

$$
\begin{aligned}
&\frac{1}{4\pi}(1-R)\left\langle u_{k_z}\middle| p\right\rangle+\alpha_{k_z}\frac{1}{4\pi}W_{u\leftarrow s}+\beta_{k_z}\frac{1}{4\pi}W_{u\leftarrow a}=A_{k_z}+B_{k_z},\\
&\frac{1}{4\pi}(1-R)\left\langle l_{k_z}\middle| p\right\rangle+\alpha_{k_z}\frac{1}{4\pi}W_{l\leftarrow s}+\beta_{k_z}\frac{1}{4\pi}W_{l\leftarrow a}=C_{k_z}+D_{k_z},\\
&\frac{1}{4\pi}T\left\langle u_{k_\zeta}\middle| p\right\rangle+\sigma_{k_\zeta}\frac{1}{4\pi}W_{u\leftarrow s}+\tau_{k_\zeta}\frac{1}{4\pi}W_{u\leftarrow a}=A_{k_z}e^{i\kappa_x w}+B_{k_z}e^{-i\kappa_x w},\\
&\frac{1}{4\pi}T\left\langle l_{k_\zeta}\middle| p\right\rangle+\sigma_{k_\zeta}\frac{1}{4\pi}W_{l\leftarrow s}+\tau_{k_\zeta}\frac{1}{4\pi}W_{l\leftarrow a}=C_{k_\zeta}e^{i\kappa_x w}+D_{k_\zeta}e^{-i\kappa_x w}.
\end{aligned}
\tag{S7}
$$

All remaining equations in Eq. (S5) except for the first and second lines become

$$
\begin{aligned}
\alpha_{k_\zeta}&=-\frac{1}{2}\left(A_{k_\zeta}-B_{k_\zeta}\right)W_{s\leftarrow u}-\frac{1}{2}\left(C_{k_z}-D_{k_z}\right)W_{s\leftarrow l}\\
\beta_{k_\zeta}&=-\frac{1}{2}\left(1-\frac{k_\zeta^2}{p_z^2}\right)^{-1}\left(A_{k_\zeta}-B_{k_\zeta}\right)W_{a\leftarrow u}-\frac{1}{2}\left(1-\frac{k_\zeta^2}{p_z^2}\right)^{-1}\left(C_{k_z}-D_{k_z}\right)W_{a\leftarrow l}\\
\sigma_{k_\zeta}&=\frac{1}{2}\left(A_{k_\zeta}e^{i\kappa_x w}-B_{k_\zeta}e^{-i\kappa_x w}\right)W_{s\leftarrow u}+\frac{1}{2}\left(C_{k_z}e^{i\kappa_x w}-D_{k_z}e^{-i\kappa_x w}\right)W_{s\leftarrow l}\\
\tau_{k_\zeta}&=\frac{1}{2}\left(1-\frac{k_\zeta^2}{p_z^2}\right)^{-1}\left(A_{k_\zeta}e^{i\kappa_x w}-B_{k_\zeta}e^{-i\kappa_x w}\right)W_{a\leftarrow u}+\frac{1}{2}\left(1-\frac{k_\zeta^2}{p_z^2}\right)^{-1}\left(C_{k_z}e^{i\kappa_x w}-D_{k_z}e^{-i\kappa_x w}\right)W_{a\leftarrow l}
\end{aligned}
\tag{S8}
$$

Here, $W_{l\leftarrow s}\equiv 2\sqrt{\pi}$, $W_{u\leftarrow s}\equiv\sqrt{\pi}\left(e^{ik_z h}+e^{-ik_z h}\right)$, $W_{u\leftarrow a}\equiv\sqrt{\pi}\left(\frac{k_z}{p_z}\left(e^{ik_z h}+e^{-ik_z h}\right)+e^{ik_z h}-e^{-ik_z h}\right)$, and $W_{l\leftarrow a}\equiv-\sqrt{\pi}\,\frac{2k_z}{p_z}$. $W_{v_1\leftarrow v_2}$ is the complex conjugation of $W_{v_2\leftarrow v_1}$ for any v_1 and v_2. Equation (S8) can be plugged into Eq. (S7), and this gives rise to four coupled equations for coefficients *A*, *B*, *C*, and *D* such that

$$
\begin{aligned}
&\left(m_{1,1}+2\right)A_{k_z}-\left(m_{1,1}-2\right)B_{k_z}+m_{1,2}C_{k_z}-m_{1,2}D_{k_z}=\frac{1}{2\pi}(1-R)\left\langle u_{k_z}\middle| p\right\rangle,\\
&-\left(m_{1,1}-2\right)A_{k_z}e^{ik_x w}+\left(m_{1,1}+2\right)B_{k_z}e^{-ik_x w}-m_{1,2}C_{k_z}e^{ik_x w}+m_{1,2}D_{k_z}e^{-ik_x w}=\frac{1}{2\pi}T\left\langle u_{k_z}\middle| p\right\rangle,\\
&m_{2,1}A_{k_z}-m_{2,1}B_{k_z}+\left(m_{2,2}+2\right)C_{k_z}-\left(m_{2,2}-2\right)D_{k_z}=\frac{1}{2\pi}(1-R)\left\langle l_{k_z}\middle| p\right\rangle,\\
&-m_{2,1}A_{k_z}e^{ik_x w}+m_{2,1}B_{k_z}e^{-ik_x w}-\left(m_{2,2}-2\right)C_{k_z}e^{ik_x w}+\left(m_{2,2}+2\right)D_{k_z}e^{-ik_x w}=\frac{1}{2\pi}T\left\langle l_{k_z}\middle| p\right\rangle,
\end{aligned}
\tag{S9}
$$

where $m_{i,j}$ is defined by

$$m_{i,j} \equiv \frac{1}{4\pi}\left[W_{v_i \leftarrow s} W_{s \leftarrow v_j} + \left(1 - \frac{k_z^2}{p_z^2}\right)^{-1} W_{v_i \leftarrow a} W_{a \leftarrow v_j} \right], \;\; v_1 \equiv u, \;\; v_2 \equiv l. \tag{S10}$$

Apparently, Eq. (S9) can be simplified to a matrix equation such that

$$MV = \begin{bmatrix} M_{11} & M_{12} \\ M_{21} & M_{22} \end{bmatrix} V = F, \tag{S11}$$

with M_{mn} the 2 × 2 submatrix, and V and F the 4 × 1 column vectors defined as

$$\begin{aligned} M_{ij} &\equiv \begin{bmatrix} m_{i,j} + 2\delta_{ij} & -m_{i,j} + 2\delta_{ij} \\ \left(-m_{i,j} + 2\delta_{ij}\right) e^{ik_x w} & \left(m_{i,j} + 2\delta_{ij}\right) e^{-ik_x w} \end{bmatrix}, \; V^T \equiv \begin{bmatrix} A_{k_z} & B_{k_z} & C_{k_z} & D_{k_z} \end{bmatrix}, \\ F^T &\equiv \frac{1}{2\pi}\Big[(1-R)\langle u_{k_z} | p \rangle \quad T\langle u_{k_z} | p \rangle \quad (1-R)\langle l_{k_z} | p \rangle \quad T\langle l_{k_z} | p \rangle \Big]. \end{aligned} \tag{S12}$$

Explicit forms of $m_{i,j}$ under the first BA are given by

$$\begin{aligned} m_{1,1} &= 1 + \frac{1}{2}\frac{k_z p_z}{k_z^2 - p_z^2}\left[\frac{k_z}{p_z}\left(e^{2ik_z h} + e^{-2ik_z h}\right) + e^{2ik_z h} - e^{-2ik_z h} \right], \\ m_{1,2} &= -\frac{1}{2}\frac{p_z^2}{k_z^2 - p_z^2}\left(e^{ik_z h} + e^{-ik_z h} + \frac{k_z}{p_z}\left(e^{ik_z h} - e^{-ik_z h}\right)\right), \\ m_{2,1} &= -\frac{1}{2}\frac{p_z^2}{k_z^2 - p_z^2}\left(e^{ik_z h} + e^{-ik_z h} + \frac{k_z}{p_z}\left(e^{ik_z h} - e^{-ik_z h}\right)\right) = m_{1,2}, \\ m_{2,2} &= \frac{2k_z^2 - p_z^2}{k_z^2 - p_z^2}. \end{aligned} \tag{S13}$$

4. Reflection and tunneling coefficients

The four coefficients A, B, C, and D can be directly obtained from Eq. (S11), and then we plug them into the first and second lines of Eq. (S5). Let us define a new matrix N such that

$$N \equiv M^{-1}. \tag{S14}$$

Also, let $n_{i,j}$ be the elements of N. Then, after some manipulations, one can show that the first and second lines of Eq. (S5) can be rewritten as

$$\begin{aligned} &(1+R) = (1-R) I_1 + T I_2, \\ &T = -(1-R) I_2 - T I_1, \end{aligned} \tag{S15}$$

where two coupling strengths I_1 and I_2 are defined as

$$\begin{aligned} I_1 &\equiv \frac{1}{2\pi} \frac{1}{\langle p | p \rangle} \int_{-\infty}^{\infty} dk_z \left[\langle p | u_{k_z} \rangle P_{11} + \langle p | l_{k_z} \rangle P_{31} \right], \\ I_2 &\equiv \frac{1}{2\pi} \frac{1}{\langle p | p \rangle} \int_{-\infty}^{\infty} dk_z \left[\langle p | u_{k_z} \rangle P_{12} + \langle p | l_{k_z} \rangle P_{32} \right], \end{aligned} \tag{S16}$$

with additional factors P_{ij} given by

$$\begin{aligned} P_{11} &\equiv \frac{k_x}{p_x} \left[\langle u_{k_z} | p \rangle (n_{1,1} - n_{2,1}) + \langle l_{k_z} | p \rangle (n_{1,3} - n_{2,3}) \right], \\ P_{31} &\equiv \frac{k_x}{p_x} \left[\langle u_{k_z} | p \rangle (n_{3,1} - n_{4,1}) + \langle l_{k_z} | p \rangle (n_{3,3} - n_{4,3}) \right], \\ P_{12} &\equiv \frac{k_x}{p_x} \left[\langle u_{k_z} | p \rangle (n_{1,2} - n_{2,2}) + \langle l_{k_z} | p \rangle (n_{1,4} - n_{2,4}) \right], \\ P_{32} &\equiv \frac{k_x}{p_x} \left[\langle u_{k_z} | p \rangle (n_{3,2} - n_{4,2}) + \langle l_{k_z} | p \rangle (n_{3,4} - n_{4,4}) \right]. \end{aligned} \tag{S17}$$

Finally, we arrive at the reflection and tunneling coefficients as follows:

$$R = \frac{I_1^2 - 1 - I_2^2}{(I_1 + 1)^2 - I_2^2}, \; T = \frac{-2I_2}{(I_1 + 1)^2 - I_2^2}. \tag{S18}$$

5. Numerical results on the resonant tunneling of 2DSPs with lossy system

The impact of system's losses is numerically demonstrated in Fig. S2. To mimic the case of graphene plasmon polaritons, we set λ_0 = 6 μm. We can see that the loss in the 2D crystal plays an

important role in the amplitude of resonant tunneling, but it has minimal impact on the reflection phase as it does not alter the resonance condition. On the other hand, at $\lambda_0 = 6$ μm, the loss in the gold nano-plates introduces a negligibly small change in both the resonant tunneling condition and amplitude, suggesting that metallic loss of the gold nano-plates does not significantly affect the tunneling (T) and reflection (R) coefficients.

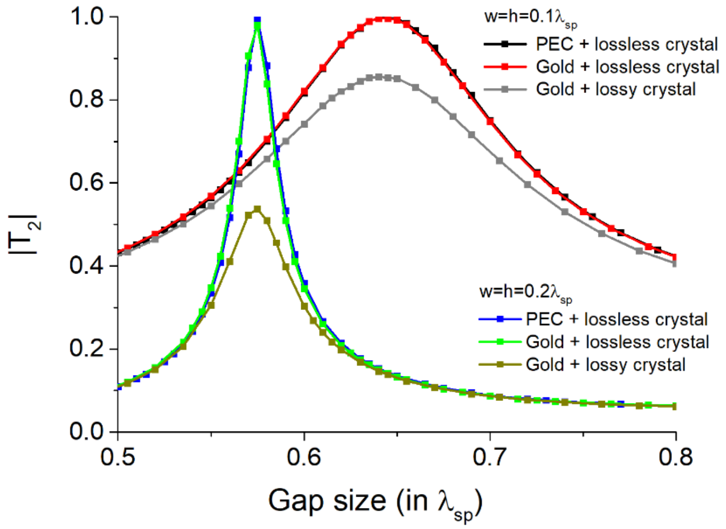

Fig. S2. Resonant tunneling of 2DSPs with losses, supplementing Fig. 4(a) in the main text. The loss of 2D crystal is implemented by assuming the momentum relaxation time of 0.5 ps in the Kubo's theory of graphene's conductivity [6, 7]. In our case, this is equivalent to the effective permittivity of $-127.3 + 1.6i$ with 0.3 nm thickness.

References

[1] A. Alù and N. Engheta, J. Opt. Soc. Am. B **23**, 571 (2006)

[2] J. H. Kang, S. Wang *et al*., Nano Lett. **17**, 1768 (2017).

[3] S. Lee and J. H. Kang, Nanophotonics **12**, 2573 (2023).

[4] S. Lee and J. H. Kang, Results in Physics **57**, 107400 (2024).

[5] D. Marcuse, *Theory of Dielectric Optical Waveguides* (Academic, 1974).

[6] L. A. Falkovsky and S. S. Pershoguba, Phys. Rev. B **76**, 153410 (2007).

[7] V. P. Gusynin *et al*., J. Phys. Condens. Matt. **19**, 026222 (2007).
